# Effect of Xanthan–Chitosan Microencapsulation on the Survival of *Lactobacillus acidophilus* in Simulated Gastrointestinal Fluid and Dairy Beverage

**DOI:** 10.3390/polym10060588

**Published:** 2018-05-28

**Authors:** Guowei Shu, Yunxia He, Li Chen, Yajuan Song, Jili Cao, He Chen

**Affiliations:** 1 School of Food and Biological Engineering, Shaanxi University of Science and Technology, Xi’an 710021, China; skyheyxkd@gmail.com (Y.H.); skdsongyj@gmail.com (Y.S.); chenhe419@gmail.com (H.C.); 2 College of Food Engineering and Nutritional Science, Shaanxi Normal University, Xi’an 710119, China; 3 Department of Research and Development, Xi’an Oriental Dairy Co., Ltd., Xi’an 710027, China; xiandfcjl@gmail.com

**Keywords:** xanthan–chitosan, encapsulation, *Lactobacillus acidophilus*, gastrointestinal fluid, bile salt, storage stability

## Abstract

*Lactobacillus acidophilus* was encapsulated in xanthan–chitosan (XC) and xanthan–chitosan–xanthan (XCX) polyelectrolyte complex (PEC) gels by extrusion method. The obtained capsules were characterized by X-ray diffraction and FTIR spectroscopy. The effects of microencapsulation on the changes in survival and release behavior of the *Lactobacillus acidophilus* during exposure to simulated gastric fluid (SGF) and simulated intestinal fluid (SIF) were studied. Encapsulated *Lactobacillus acidophilus* exhibited a significantly higher resistance to SGF and SIF than non-encapsulated samples. In addition, the viability of free and immobilized cells of *Lactobacillus acidophilus* incorporated into dairy beverages was assessed for 21 days both at room temperature and in refrigerated storage. The results indicated that xanthan–chitosan–xanthan (XCX) and xanthan–chitosan (XC) significantly (*p* < 0.05) improved the cell survival of *Lactobacillus acidophilus* in yogurt during 21 days of storage at 4 and 25 °C, when compared to free cells.

## 1. Introduction

Functional foods with the addition of living probiotic microorganisms, which are claimed to have positive effects on the host, have become an important trend in the food industry in recent years. Probiotics are described as “live microorganisms which when administered in adequate amounts confer a health benefit on the host” [1,2]. Probiotics are believed to play a beneficial role in the ecosystem of the human intestinal tract, which affects human health by improving the gut microbiota balance and defenses against pathogens. To exert benefits on human health, the concentration of live probiotic bacteria should be approximately 10^7^ CFU/mL in the product at the time of consumption. However, probiotics are vulnerable to adverse conditions, such as oxidative stress, temperature, pH variations, and osmotic stress during the entire food processing operation, including storage and gastrointestinal transit, resulting in a significant reduction in viable count [3,4,5].

Microencapsulation is the envelopment of core material in a coating that protects it from adverse environments, and has been used widely in the food industry [6,7]. Many polymer materials, such as pectin, alginate, carrageenan, chitosan, whey, gelatin, and starch, have been used for bacteria microencapsulation, as reported in several studies [8,9]. Among various bio-polymeric encapsulation systems, the xanthan–chitosan mixture has been identified as a high-potential hydrogel system for targeted delivery and controlled release of encapsulated products for oral administration.

**Figure 1:**
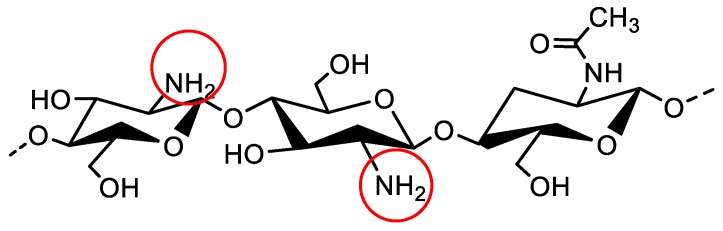
Chemical structure of chitosan.

Chitosan (Figure 1), partially N-acetylated poly-β-(1-4)-d-glucosamine, is the only naturally cationic polysaccharide, obtained by partial deacetylation of chitin. The unique properties of chitosan arise from its amino groups, which carry positive charges at pH values below 6.5, the polycationic properties of which facilitate the preparation of various polyelectrolyte complexes with natural polyanions [10,11,12].

**Figure 2:**
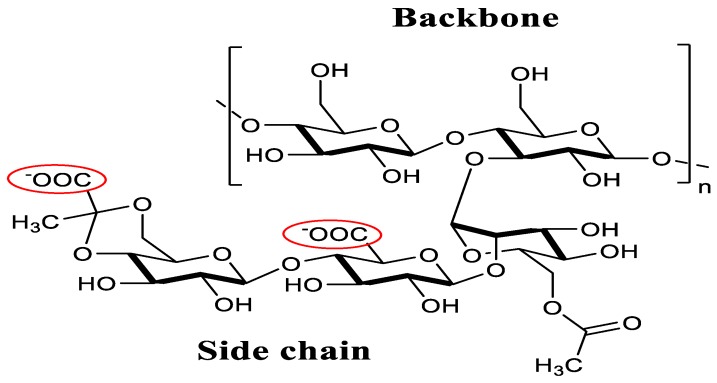
Chemical structure of xanthan.

Xanthan gum (Figure 2) is a microbial exopolysaccharide, obtained from submerged aerobic fermentation of a pure culture of the genus *Xanthomonas campestris*. It consists of a cellulosic backbone, namely (1,4)-β-d-glucopyranose glucan, with a trisaccharide side chain, namely (3,1)-α-d-mannopyranose-(2,1)-β-d-glucuronic acid-(4,1)-β-d-mannopyranose, on every second glucose residue [13], which is considered an anionic polyelectrolyte. Xanthan gum and chitosan form a three-dimensional network through strong electric interactions between the amine (chitosan) and carboxylic (xanthan) groups, which can absorb much more water than their own weight [14,15].

In our previous research, the effects of chitosan concentration, xanthan concentration, and a xanthan–*Lactobacillus acidophilus* mixture (XLM)/chitosan on *Lactobacillus acidophilus* microcapsules have been studied by response surface methodology [16]. The aim of this study was to assess the ability of an encapsulation system (XC and XCX) to improve the survival of *Lactobacillus acidophilus* during exposure to simulated gastrointestinal solution, bile salt solution, and in a dairy beverage at 4 and 25 °C.

## 2. Materials and Methods

### 2.1. Strains

*Lactobacillus acidophilus*, isolated from a yogurt starter (Synbiotech Biotechnology Co., Ltd. NanJing, China), was preserved in the lab of School of Food and Biological Engineering, Shaanxi University of Science and Technology, were inoculated in Man Rogosa Sharpe (MRS) broth and incubated at 37 °C for 24 h. The cells were harvested by centrifugation at 10,000 rpm for 15 min and re-suspended in 0.9% sterilized saline water.

### 2.2. Microencapsulation Procedure

Xanthan (Zhongxuan Biological Chemistry Co., Ltd., Shandong, China) with a molecular weight (*M_W_*) of 1020 kDa and a degree of substitution per side chain of 0.73 and 0.70 for acetyl and pyruvate groups, respectively (reported by supplier), was dissolved in deionized water (0.76% *w*/*v*). Then the solution was sterilized with moist heat at 110 °C for 10 min, cooled, and centrifuged at 7000 r for 10 min. Chitosan, supplied by Xingcheng Biological Co., Ltd. (Jiangsu, China), with a *M_W_* of 370 kDa and degree of acetylation of 17% (reported by supplier) was dissolved into 1 mol·L^−1^ stirring HCL solution (0.68% *w*/*v*, pH 5.5).

A *Lactobacillus acidophilus* culture was dispersed in xanthan solution (1:10). The mixture was injected through a manually operated syringe with 0.45 mm cannula into chitosan solution. After 40 min magnetic stirring, the microcapsules (XC) were formed. The wet capsules were then filtered through 160 mm gauze, followed by washing with sterilized saline water three times, after which monolayer (XC) *Lactobacillus acidophilus* microcapsules was obtained. Then 0.1% (*w*/*v*) xanthan solution was added to the XC microcapsules; after being stirred for 30 min on the magnetic stirrer, the double microcapsules (XCX) were prepared for further analysis.

### 2.3. Viable Counts and Encapsulation Yield

The plate counting method was used to measure the viable count of *Lactobacillus acidophilus*. After being appropriately diluted by sterile saline solution, 0.1 mL bacterial suspension was inoculated to the solid culture medium. The viable count was determined after the bacterium was incubated in MRS at 37 °C for 48 h.

The encapsulation yield (EY) was assessed using the method described by Shu [16]: 1 g of microcapsules were subjected to 10 mL simulated intestinal fluid, after being oscillated at 37 °C for 40 min under 210 rpm. The microencapsulation yield was calculated according to Equation (1):

(1) $${\mathrm{EY}=(N}_{1}\cdot M)/\left(N_{0}\cdot V_{0}\right)\;\times 100\%$$

where N_1_ (CFU·mL^−1^) was viable counts released from microcapsules after being subjected to simulated intestinal fluid. *M* (g) was the weight of the wet microcapsule. *N*_0_ (CFU·mL^−1^) was the initial viable counts in the cell suspension. *V*_0_ (mL) was the volume of cell suspension for microencapsulation.

### 2.4. Characterization of the Capsules

#### 2.4.1. X-ray Diffraction (XRD)

Samples were filled into a sample holder and exposed to CuKα radiation in an X-ray powder diffractometer (D8, Bruker AXS, Karlsruhe, Germany). Each sample was scanned in continuous mode at a scanning rate of 4°/min with the diffraction angle 2 h from 3°–50°.

#### 2.4.2. Fourier Transform Infrared Spectroscopy

Fourier transform infrared (FTIR) spectra of some samples, including chitosan, xanthan, and xanthan–chitosan beads were recorded using a FTIR spectrophotometer (Thermo Scientific Nicolet iS50, Waltham, MA, USA). The samples were crushed with potassium bromide to make pellets. The FTIR spectra were scanned in the range of 400–4000 cm^−1^, and the resolution was 4 cm^−1^.

### 2.5. Preparation of Simulated Gastric Fluid and Simulated Intestinal Fluid

Simulated gastric fluid (SGF) was prepared by dissolving the mixture of 16.4 mL of HCl (0.1 mol·L^−1^) and 10g pepsin in 1000 mL deionized (DI) water and adjusting the pH to 1.2. To prepared simulated intestinal fluid (SIF), 6.8 g of KH_2_PO_4_ (0.1 mol·L^−1^) was dissolved in 250 mL of deionized water, followed by adding NaOH (0.2 mol·L^−1^) until the pH reached 6.8. Trypsin (10 g) was dissolved in 400 mL deionized water and mixed with the above-mentioned KH_2_PO_4_ solution. The mixture was diluted with deionized water to 1 L. A membrane filter (0.45 µm) was used to sterilize the mixture.

### 2.6. Release Profile of Lactobacillus acidophilus

To investigate the release behavior of *Lactobacillus acidophilus* from the xanthan–chitosan capsules in gastro-intestinal fluid, 9 mL of brown milk beverage containing 1 g of *Lactobacillus acidophilus* microcapsules or 1 mL free *Lactobacillus acidophilus* suspension was incorporated into 40 mL SGF (pH = 1.5). The sample was incubated at 37 °C for 0, 0.5, 1, 1.5, and 2 h while shaking at 110 rpm. Subsequently, it was continuously transferred into SIF (pH of 6.8) for another 2 h. wet microcapsules were collected every 30 min and washed with sterilized saline water. They were then used for the enumeration of the viable count. A spectrophotometer was employed for release study. A 2.0 mL aliquot was collected and the absorbance at 600 nm was determined in the stipulated time (every 30 min).

### 2.7. Storage Stability

Microcapsules of 1 g XC and 1 g XCX were each incorporated into respective 9 mL brown milk beverages supplied by Xi’an Oriental Dairy Co., Ltd. (Xi’an, China). A control group was done by adding a 1 mL free *Lactobacillus acidophilus* suspension to an additional 9 mL brown milk beverage. Viable counts, acidity, and pH were determined in the stipulated time (0, 1, 3, 7, 14, and 21 d) at 4 and 25 °C respectively. The viable count was determined by the plate count method described in [17]. The data were expressed as mean ± standard deviation (SD) of three independent experiments. The acidity (°T) was determined by the method of neutralization titration, according to the GB5413.34-2010 (“National food safety standard: Determination of acidity in milk and milk products”). The pH of each sample was recorded using a pH-meter (pHS-3C Shanghai Precision Scientific Instrument Co., Ltd., Shanghai, China). Experiments were conducted in triplicates.

### 2.8. Statistical Analysis

The mean values and standard deviation were calculated from the data obtained with triplicate trials. Results are presented as mean ± standard deviation (SD) of replicated determinations.

## 3. Results and Discussion

### 3.1. Characterization of the Capsules

**Figure 3:**
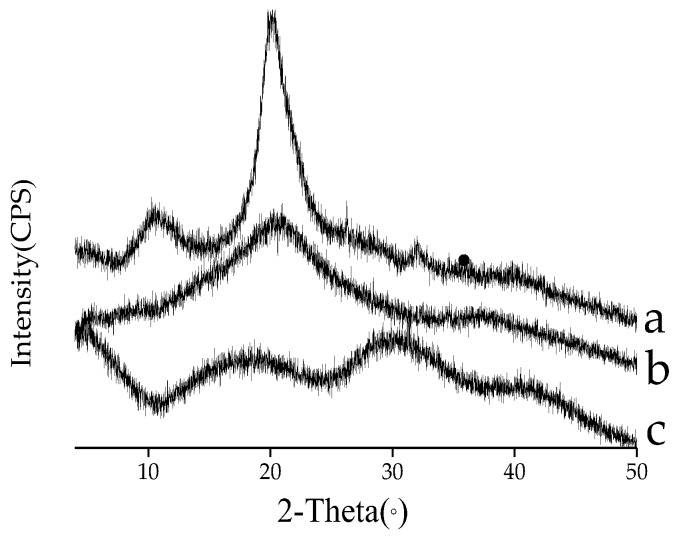
X-ray diffractogram of (**a**) chitosan, (**b**) xanthan, and (**c**) xanthan–chitosan capsules.

XRD is a useful tool for studying the crystal lattice arrangement of the sample [18]. The X-ray diffractograms of chitosan, xanthan, and xanthan–chitosan capsules were presented in Figure 3. The XRD pattern of chitosan displayed two strong crystalline peaks at around 10° and 20°, indicating the semi-crystalline nature of chitosan. Similar results have been reported by other researchers [19,20]. The semi-crystalline nature of chitosan could be attributed to the presence of a large number of amino and hydroxyl groups on its structure, which can form strong inter- and intramolecular hydrogen bonds [21]. The diffraction pattern of xanthan gum did not have peaks associated with a short-range organization of the chains, which were characterized by a broad peak within the xanthan gum’s XRD graph, indicating a typically amorphous nature. This might be due to the existence of side chains in type β-d-mannose, (1,4)-β-d-glucuronic acid, as well as (1,2)-α-d mannose in the chemical structure of the xanthan gum, where the packaging of the chains is difficult [11]. Compared to the pure biopolymers, a different profile was observed in the diffraction analysis of the XC capsule. The sharp peak associated with the most crystalline phase of chitosan was not observed at 20° for the capsule, the crystalline peaks observed in xanthan disappeared. This result indicated that the crystalline structures of xanthan and chitosan were disrupted after being combined together in the formulation of capsules, impeding the formation of hydrogen bonding between the amino and hydroxyl groups.

**Figure 4:**
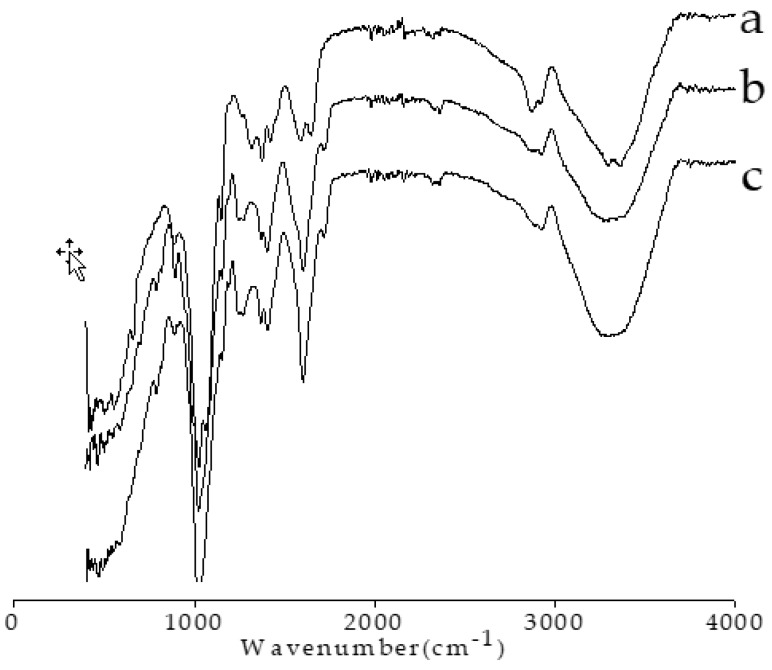
FTIR spectra of (**a**) chitosan, (**b**) xanthan–chitosan capsule, and (**c**) xanthan.

The FTIR spectra of the chitosan, xanthan, and XC capsules are shown in Figure 4. The spectrum of pure chitosan powder (Figure 4a), has shown characteristic absorption bands at 1644 (amide I), 1581 (NH_2_), and 1382 cm^−1^, the last of which was attributed to the distorting vibration of C–CH_3_ (amide II). Xanthan showed characteristic bands at 1043 (C–O linkage from alcohol group), 1720 (characteristic to acetate and pyruvate groups), 2929 (C–H stretching), and 3315 cm^−1^ (–OH stretching). The XC capsules showed similar spectra to that of xanthan: a broad band centered at 3301 cm^−1^, which can be assigned to –OH and –NH stretching modes involved extensively in inter- and intramolecular hydrogen bonds of the capsule components (chitosan, xanthan gum).

### 3.2. Viability of Lactobacillus acidophilus Microcapsules in Gastrointestinal Fluid

**Figure 5:**
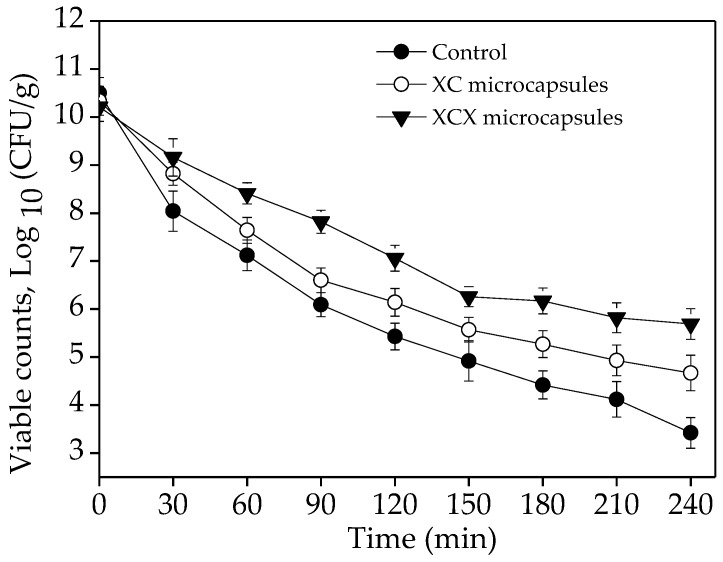
Comparison of the survival of free and encapsulated *Lactobacillus acidophilus* cells in the brown milk beverage, during 2 h exposure to SGF and 2 h exposure to SIF. The error bars represent standard deviations of the mean (*n* = 3).

Probiotics exhibit poor viability in gastric juice after oral application, which can compromise their efficacy. From the functional point of view, the main purpose of microencapsulation is to increase the survival of *Lactobacillus acidophilus* cells during exposure to gastrointestinal conditions after consumption of a probiotic milk beverage, which is necessary for the probiotic activity in vivo. In this study, *Lactobacillus acidophilus* microcapsules (XC and XCX) were subjected to SGF (pH = 1.5) for 2 h and SIF (pH = 6.8) for another 2 h. The entrapment of *Lactobacillus acidophilus* cells with xanthan–chitosan complex significantly protected the cells in gastro-intestinal fluid (Figure 5). All probiotic organisms tested showed a trend of decreasing viability when exposed to acidic conditions for 2 h. In addition, a severe decline in survival was exhibited in free *Lactobacillus acidophilus* cells. The initial cell count was approximately 10.21–10.5 CFU·mL^−1^ in the brown milk beverage. After subjected to SGF for 1 h, non-encapsulated free cells suffered a 3.38-log reduction whereas survival loss was approximately 2.7 and 1.8 log in the case of XC microcapsules and XCX microcapsules, respectively. After being dispersed in SGF for 2 h, the XCX and XC microcapsules showed an 1.92 and 0.87 log preservation of *Lactobacillus acidophilus* cells over the freely suspended cells. Free cells were drastically reduced to 5.43 log CFU·mL^−1^ at the end of 2 h in SGF. The encapsulated cells exhibited significantly improved survival, and the XCX microcapsules showed a positive effect on the survival of encapsulated cells, which proved that XC and XCX microcapsules showed resistance to gastric acid. Similarly, Chandramouli [22] reported that alginate significantly increases the viable count of *Lactobacillus acidophilus* at pH 2.0. The protective effect of the XC and XCX matrix could be explained by the following: when the XC gel was dispersed in the acidic solution, the carboxyl group was deionized, while the amino groups were positively charged, so electrostatic linkage between the two functional groups disappeared, resulting in the increase of osmotic pressure and swelling in the XC gels, where water permeated into the capsules to buffer acidic compounds [23]. Hence, *Lactobacillus acidophilus* encapsulated in XC microcapsules showed certain resistance to low-pH gastric fluid, and XCX strengthened the protective effect.

The viability of encapsulated and non-encapsulated cells was compared during incubation in SIF. The results indicated that the viability of all free cells, XC-encapsulated cells and XCX-encapsulated cells decreased by approximately 2.01, 1.47, and 1.37 log (CFU·g^−1^), respectively. It could be observed that there were approximately 1.01 log cycle losses in number of cells of free *Lactobacillus acidophilus* after being subjected to SIF for 1 h, while the amounts of XC and XCX *Lactobacillus acidophilus* microcapsules decreased by 0.87 log and 0.89 log CFU·g^−1^. A severe decline (2.01 log CFU·g^−1^) of viability was exhibited in free *Lactobacillus acidophilus* after incubation for 2 h when compared to the XC and XCX *Lactobacillus acidophilus* microcapsules. The XC microcapsules showed 0.54 log preservation over the free cells, and the XCX microcapsules exhibited 0.64 log preservation over the free cells. Therefore, the encapsulation appeared to be effective in protecting probiotic cells during passage through the human stomach. These results could be explained by an ion exchange reaction between the beads and bile salt, which could decrease the network porosity. The lower porosity and thicker double-layer structure might limit the diffusion of bile salt into the microcapsules, resulting in the reduction of bacterial stress. Similarly, Mandal [24] reported that free cells of *Lactobacillus casei* NCDC-298 showed decreased viability when the cells were exposed to 1% and 2% bile salts for 12 h, from 9.45 to 7.29 log CFU mL^−1^ and from 9.34 to 5.60 log CFU mL^−1^, respectively. The viability of the probiotics improved after immobilization in alginate. In addition, much higher numbers of living cells reached the intestine via encapsulation, which was essential to confer more desirable health benefits.

### 3.3. Release of Lactobacillus acidophilus from Capsules in Gastrointestinal Fluid

**Figure 6:**
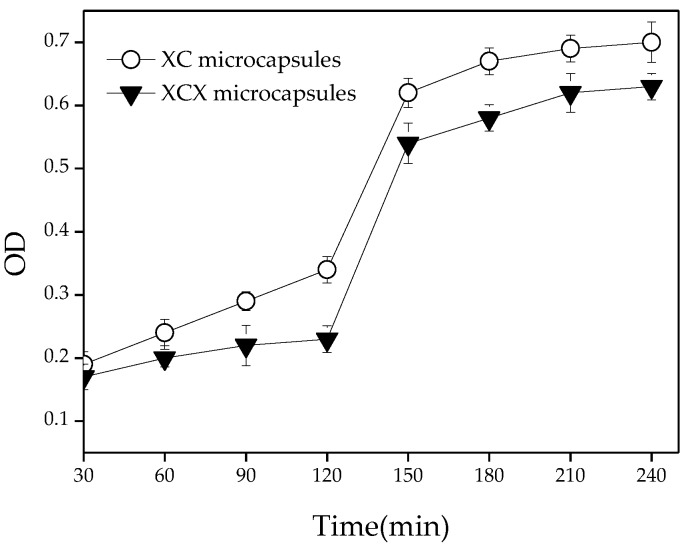
The release profile of *Lactobacillus acidophilus* microcapsules during 2 h exposure to SGF and 2 h exposure to SIF. The error bars represent standard deviation of means (*n* = 3).

The release of cells from microcapsules in the colon is essential for the growth and colonization of probiotics. An efficient release of viable and metabolically-active cells in the intestine is one of the main aims of microencapsulation. The release characteristics of encapsulated *Lactobacillus acidophilus* from the XC and XCX microcapsules was investigated. Capsule samples (XC and XCX) were treated with SGF and SIF to control the continuous release characteristics in the gastrointestinal tract. Figure 6 represented the cells released from the XC and XCX microcapsules during exposure to SGF and SIF. *Lactobacillus acidophilus* cells were released from capsules in both acidic (pH 1.5) and neutral (pH 6.8) conditions. Upon exposure to SIF, the release amount of *Lactobacillus acidophilus* cells from XCX microcapsules was lower than from the XC capsules. After transferring the beads from SGF to SIF, the capsule samples showed a larger amount and faster release rate of *Lactobacillus acidophilus* cells. The XC and XCX microcapsules reached maximum release after 2 h incubation in the SIF.

### 3.4. Application of Lactobacillus acidophilus Microcapsules in Dairy Beverage

**Figure 7:**
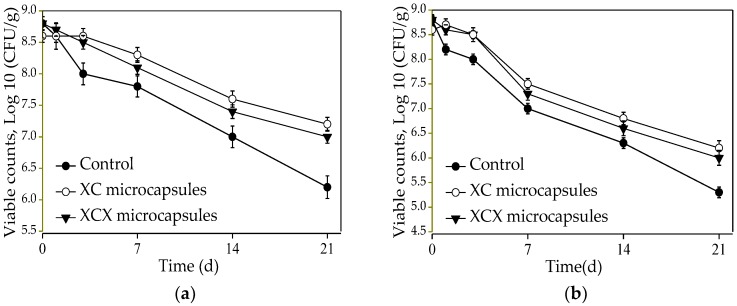
Survival of encapsulated and free *Lactobacillus acidophilus* incorporated into dairy beverage during storage at 4 °C (**a**) and 25 °C (**b**). ● Free *Lactobacillus acidophilus*; ○ XC microcapsules; ▼ XCX microcapsules. The error bars represent standard deviation of the mean (*n* = 3).

Dairy beverage is also a dairy product, as a vehicle for the supply of probiotic microcapsules. The survival of *Lactobacillus acidophilus* in the dairy beverage during 21 days of storage at either 4 °C or 25 °C is shown in Figure 7. Viable counts of free *Lactobacillus acidophilus* decreased by 2.6 log (from 8.8 to 6.2 log CFU/mL) for 21 d in the dairy beverage at 4 °C, while the viable counts of dairy drinks containing single- and double-layer *Lactobacillus acidophilus* microcapsules decreased by 1.8 log and 1.4 log CFU/g, respectively. For *Lactobacillus acidophilus* in free form, there was an obvious drop (from 8.8 to 5.3 log CFU/mL) in the number of viable cells during 21 d storage at 25 °C, while there was an approximately 2.8 log and 2.4 log decrease in the number of viable cells in the single- and double-layer *Lactobacillus acidophilus* microcapsules. The results exhibited that free and encapsulated cells had a more severe decline in their survival rate in dairy drinks at 25 °C than at 4 °C. In addition, there was a smaller loss of double-layer than single-layer microcapsules with regards to the survival of *Lactobacillus acidophilus* during 21 d storage, both at 4 and 25 °C., which proved that encapsulations played a protective role in the survival of *Lactobacillus acidophilus* during the storage of dairy drinks.

**Figure 8:**
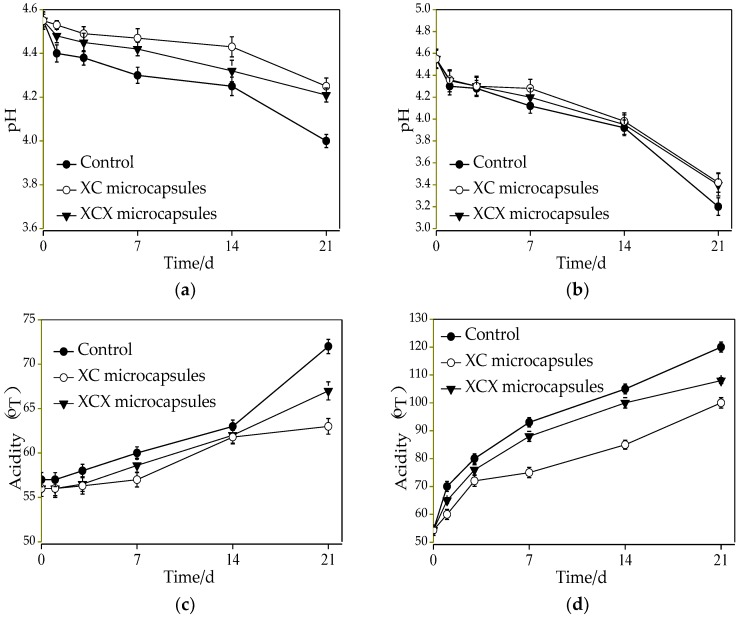
The pH of encapsulated and free *Lactobacillus acidophilus* incorporated into a dairy beverage during storage at 4 (**a**) and 25 °C (**b**); acidity of encapsulated and free *Lactobacillus acidophilus* incorporated into yogurt during storage at 4 (**c**) and 25 °C (**d**). ● Free *Lactobacillus acidophilus*; ○ XC microcapsules; ▼ XCX microcapsules. The error bars represent standard deviation of the mean (*n* = 3).

Changes of pH and acidity of all dairy drinks at 4 and 25 °C during storage are also displayed in Figure 8. Regarding the pH change in the storage of dairy drinks, the decline with free cells was from 4.55 to 4 with 21 d of storage and 4.55 to 4.21 with single-layer microcapsules; similarly, the pH varied from 4.55 to 4.25 for double-layer *Lactobacillus acidophilus* microcapsules at 4 °C. A severe decline of the pH in dairy drinks was exhibited at 25 °C. The dairy drinks containing free *Lactobacillus acidophilus* had a more accentuated decline in pH than those containing *Lactobacillus acidophilus* microcapsules. As regards the acidity, dairy drinks with single- and double-layer microcapsules increased by approximately 13 °T (from 54.3 to 67 °T) and 8 °T (from 54.3 to 63 °T), respectively, while dairy drinks with free cells increased by 18 °T within 21 d storage at 4 °C. The sharp increment of acidity in dairy drinks was shown at 25 °C due to its strong metabolism.

After a combination of survival and sensory evaluation, dairy drinks with free, XC, and XCX microcapsules were better stored at 4 °C, and the optimal storage time for dairy drinks with free, XC, and XCX microcapsules was 14, 21, and 21 d at 4 °C, respectively. The number of probiotic bacteria was maintained above 10^7^ CFU·mL^−1^ in dairy drinks during their shelf life. These results demonstrated that XC and XCX microcapsules both enhanced the stability of *Lactobacillus acidophilus* in dairy drinks during the storage period at 4 and 25 °C, respectively.

## 4. Conclusions

In this study, XC capsules were prepared by the extrusion method between two oppositely charged polysaccharides. Compared to the pure polymers, the structural and crystallinity of the capsules were changed significantly. XC and XCX capsules survived better when exposed to gastrointestinal fluid compared to non-encapsulated cells. The encapsulated cells showed a greater release in SIF than SGF. In addition, the considerable protection effect on viability of *Lactobacillus acidophilus* between XC and XCX microcapsules was achieved in the dairy beverage. The effective encapsulation system (XC and XCX) resulted in a longer storage period at both 4 and 25 °C, in comparison to the samples supplemented with free cells, which provide an experimental basis for a better application of probiotics in the development of functional foods.
